# Black Esophagus: A Case of Acute Esophageal Necrosis in a Recovering Alcoholic

**DOI:** 10.7759/cureus.98024

**Published:** 2025-11-28

**Authors:** Muhammad Sohaib Siddique, Muhammad Zahid Siddique, Shahid Siddique, Azhar Waseem

**Affiliations:** 1 Emergency Medicine, Din Medical Complex, Burewala, PAK; 2 Otolaryngology, Din Medical Complex, Burewala, PAK; 3 Surgery, Din Medical Complex, Burewala, PAK; 4 Gastroenterology, Din Medical Complex, Burewala, PAK

**Keywords:** acute esophageal necrosis, acute gastrointestinal bleed, alcoholism, black esophagus, hematemesis, upper gi endoscopy

## Abstract

Acute esophageal necrosis (AEN), often called black esophagus, is a rare but serious entity characterized by circumferential black discoloration of the esophageal mucosa. It typically presents in debilitated individuals but can also manifest following an acute insult in otherwise healthy patients. We describe a 33‑year‑old man with alcohol dependence who presented with hematemesis and coffee‑ground vomiting after a binge. Urgent endoscopy revealed extensive black necrotic lesions from the mid to distal esophagus. Conservative treatment with IV proton pump inhibitors (PPIs), fluid resuscitation, and supportive care led to gradual mucosal regeneration, confirmed on serial endoscopies. The patient recovered completely with conservative management and remained asymptomatic at the six-month follow-up. Clinicians should consider AEN in patients with upper GI bleeding, especially in the context of alcohol abuse or ischemia. Early diagnosis and prompt management can achieve full recovery.

## Introduction

Acute esophageal necrosis (AEN), often referred to as “black esophagus,” is an uncommon but severe condition characterized by a striking black discoloration of the esophageal mucosa, most prominently affecting the distal third with a sharp stop at the gastroesophageal junction. The entity was first described by Goldenberg and colleagues in 1990 [1], and since then, fewer than a few hundred cases have been reported worldwide. The true incidence remains very low, estimated at around 0.01% to 0.28% of all upper endoscopies [2].

The exact cause of AEN is not completely understood, but it is generally accepted to result from a combination of two insults: ischemic injury to the esophageal wall and chemical damage from reflux of gastric contents into already compromised tissue [3]. Conditions that decrease mucosal perfusion, such as shock, sepsis, diabetic ketoacidosis, alcohol abuse, or malnutrition commonly associated triggers [4,5].

Patients usually present with acute upper GI bleeding, often manifesting as hematemesis or melena, and may have concurrent systemic illness or hemodynamic instability [6]. Endoscopy is diagnostic, revealing circumferential black necrotic mucosa with an abrupt transition to healthy tissue. Management is primarily supportive and directed at correcting underlying causes, stabilizing circulation, and suppressing acid exposure [7].

Although the esophageal lesion itself may resolve with appropriate care, the overall prognosis remains guarded because mortality is typically driven by the underlying critical illness rather than the necrosis. Reported mortality rates range between 30% and 36% [2,5]. Given its rarity and potential severity, AEN warrants prompt recognition and management to prevent complications and improve outcomes.

We report a case of a young male, a recovering alcoholic, who developed black esophagus after a binge episode, with full recovery following conservative management.

## Case presentation

A 33‑year‑old male patient presented to our emergency department in the early hours of the day with repeated coffee‑ground vomiting and one episode of fresh hematemesis. He appeared fatigued and complained of soreness after each retch. The patient had a seven‑year history of alcohol dependence, with intermittent abstinence; he admitted to heavy drinking the night before presentation after a period of sobriety. He denied melena, dysphagia, weight loss, or prior GI disease. He had no other comorbidities, was not on any regular medications, and had no relevant family history of GI or hepatic disease. On examination, he was alert but dehydrated and had tachycardia. Vital signs were as follows: pulse 130 beats per minute (bpm), blood pressure 152/96 mmHg, temperature 36.1°C, and respiratory rate 18 breaths per minute. His abdomen was soft with epigastric tenderness; no organomegaly or masses. A rectal exam showed normal-colored stool without melena. The patient’s initial laboratory findings on admission demonstrated mild anemia, elevated liver enzymes, and evidence of renal dysfunction likely due to dehydration and internal bleeding (Table 1). Elevated blood urea nitrogen/creatinine ratio was consistent with prerenal azotemia, which in this setting was likely due to volume depletion and the increased urea production that occurs with upper GI bleeding. Elevated gamma-glutamyl transferase (GGT), in the absence of other clear cholestatic findings, supported chronic alcohol use, which was consistent with the patient’s presentation and other laboratory features.

**Table 1:** The patient's laboratory findings

Laboratory Parameter	Value	Reference Range
White blood cell (10³/µL)	11.2	4.8 – 10.8
Hemoglobin (g/dL)	11.7	14.0 – 18.0
Platelets (10³/µL)	165	150 – 450
Blood urea nitrogen (mg/dL)	77	7 – 18
Creatinine (mg/dL)	1.8	0.52 – 1.23
Aspartate aminotransferase (U/L)	88	15 – 37
Alanine transaminase (U/L)	62	12 – 78
Alkaline phosphatase (U/L)	186	45 – 117
Gamma glutamyl transferase (U/L)	210	9 – 48
Total bilirubin (mg/dL)	2.1	0.1 – 1.0

A CT scan of the abdomen and thorax with contrast was performed, which showed no leak, no mediastinal collection, the presence of paraesophageal varices, and the presence of liver cirrhosis.

The patient was admitted to the high-dependency unit and managed conservatively. He was kept nil per oral, started on intravenous isotonic fluids for rehydration, and commenced on intravenous pantoprazole 80 mg bolus followed by 8 mg/hour infusion for 72 hours. He also received ondansetron, paracetamol, thiamine supplementation (100 mg IV daily), and lactulose syrup (30 mL three times daily) to prevent hepatic encephalopathy. Broad-spectrum antibiotics were withheld as there were no signs of infection or perforation. Strict hemodynamic and urine output monitoring was maintained. His hemoglobin and coagulation profile were checked serially; no transfusions were required.

Endoscopic findings

Oesophago-Gastro-Duodenoscopy 1 (OGD; Day 1)

**Figure 1:**
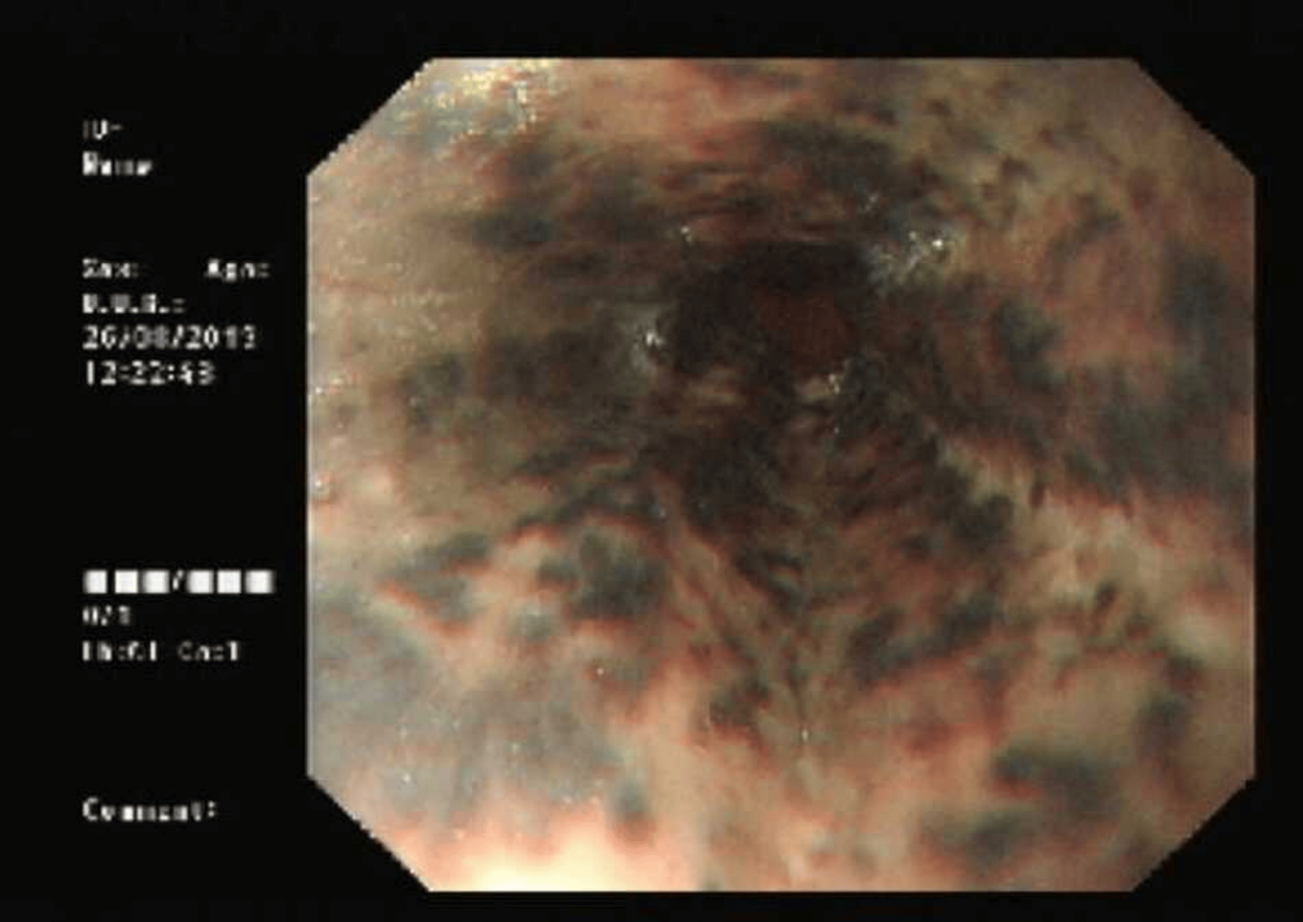
Upper GI endoscopy performed on Day 1 Circumferential black necrotic mucosa of the distal esophagus, sharply demarcated at the gastroesophageal junction; findings diagnostic of acute necrotizing esophagitis (“black esophagus”)

Under sedation (midazolam 4 mg, fentanyl 100 µg), upper GI endoscopy revealed extensive circumferential black discoloration of the esophageal mucosa extending from approximately 20 cm from the incisors to the gastroesophageal junction, with a sharp demarcation to healthy gastric mucosa. The mucosa appeared friable and hemorrhagic but without active bleeding. Mild gastritis and duodenitis were also noted. The appearance was diagnostic of AEN (Figure 1). The procedure was terminated early due to tachycardia (170 bpm).

Following endoscopy, the patient was continued on intravenous proton pump inhibitors (PPIs), IV fluids, and bowel rest. His urine output and renal function progressively improved over the next 48 hours.

OGD 2 (Day 4)

**Figure 2:**
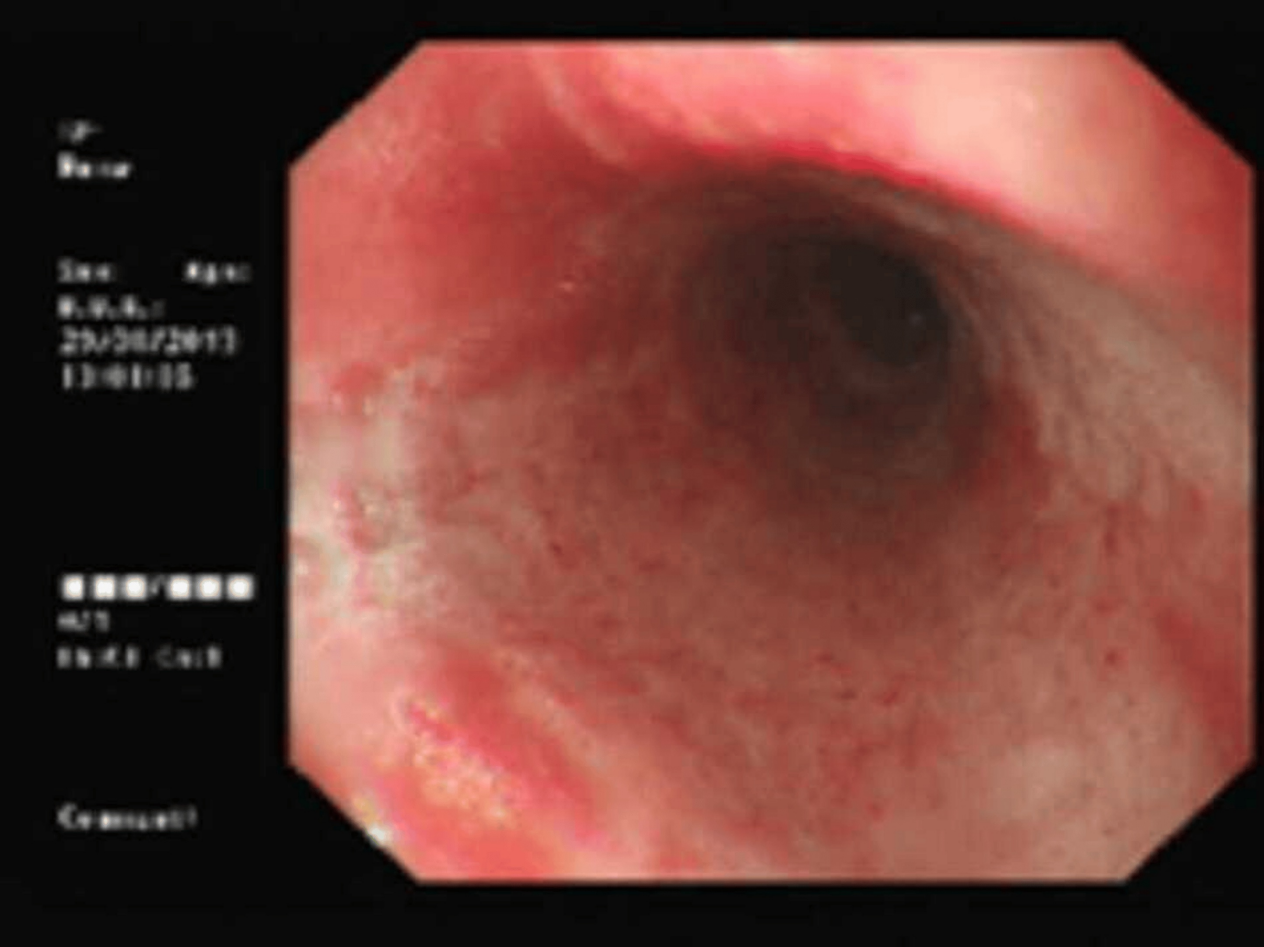
Upper GI endoscopy performed on Day 4 Resolution of black necrosis with erythematous and friable mucosa which indicates progressive recovery under conservative management.

Repeat endoscopy showed significant improvement with resolution of black discoloration and residual erythematous mucosa with erosions and minimal oozing (Figure 2). Four small esophageal varices were visualized and endoscopically band-ligated. The patient remained hemodynamically stable and afebrile. He was gradually advanced to clear fluids and then a soft diet, which he tolerated well.

OGD 3 (Day 8)

Follow-up endoscopy demonstrated healing mucosa at the banding sites with no residual necrotic tissue. Mild gastritis and duodenitis persisted. The patient was transitioned to high-dose oral pantoprazole 40 mg twice daily and sucralfate suspension 10 mL four times daily, and he received extensive alcohol cessation counseling.

His liver function tests showed improvement: total bilirubin decreased from 2.1 mg/dL to 1.2 mg/dL, and GGT decreased from 210 U/L to 140 U/L. He was discharged on Day 9 of hospitalization in stable condition with advice for strict abstinence, small, frequent meals, and regular follow-up.

At the two-week outpatient follow-up, the patient reported resolution of vomiting, improved appetite, and stable weight. He remained abstinent from alcohol and compliant with medication. At the one-month follow-up, he was asymptomatic with normalized hemoglobin (13.4 g/dL), stable renal and hepatic parameters, and no recurrence of upper GI bleeding. At the three-month follow-up (OGD 4), endoscopy revealed a small sliding hiatus hernia, mild gastritis, and duodenitis, but complete resolution of previous necrotic and ulcerative changes in the esophagus. No strictures or dysphagia were noted. At six months, he continued to remain well, abstinent from alcohol, and off acid-suppressive therapy except for occasional maintenance PPI use.

The patient's perspective was positive. He expressed gratitude for early diagnosis and emphasized that the visible improvement on serial endoscopies strengthened his motivation to maintain abstinence. A timeline of the clinical course, outcome, and management can be found in Table 2.

**Table 2:** Timeline of clinical course, management, and outcomes OGD: oesophago-gastro-duodenoscopy

Day/Period	Event/Intervention	Outcome/Findings
Day 1	Presentation with hematemesis; OGD 1 showing circumferential black necrosis of the distal esophagus	IV fluids and proton pump inhibitor initiated; bowel rest maintained
Day 4	Repeat OGD 2 performed; varices identified and banded	Resolution of black necrosis; erythematous mucosa noted
Day 8	OGD 3 follow-up	Healing ulcers at banding sites; transitioned to oral PPI and sucralfate
Day 9	Discharge	Stable; advised strict alcohol abstinence
2 Weeks	Outpatient review	Asymptomatic; normal oral intake
1 Month	Laboratory reassessment	Normalized hemoglobin and liver function
3 Months	OGD 4	Complete mucosal healing; mild gastritis
6 Months	Long-term follow-up	Sustained recovery; continued abstinence

## Discussion

AEN remains a rare but serious cause of upper GI bleeding. This condition typically arises in critically ill patients with hemodynamic compromise, sepsis, or metabolic derangements. The pathogenesis aligns with the established “two-hit” hypothesis, in which an ischemic insult to the esophageal mucosa is followed by chemical injury from gastric reflux [1,3]. Factors such as diabetes, alcohol abuse, and malnutrition further impair mucosal resilience, predisposing the esophagus to necrosis [4,5].

In our patient, the precipitating factors likely included acute alcohol binge, repeated vomiting, volume depletion, and transient hemodynamic compromise. Alcohol may also directly impair mucosal integrity and increase reflux risk. Retching can cause shear stress, mucosal tears, and local hypoperfusion. Endoscopy demonstrated circumferential black necrosis of the distal esophagus, terminating abruptly at the gastroesophageal junction; findings pathognomonic for this entity [6].

Treatment is primarily medical, involving resuscitation, acid suppression, bowel rest, and monitoring. Antibiotics are reserved for suspected perforation or infection, as secondary infection is uncommon [7,8]. Surgical intervention is rarely needed unless complications arise, such as perforation or mediastinitis. In many cases, mortality is related to comorbidities rather than the esophageal necrosis itself.

Outcomes vary according to underlying illness severity rather than the esophageal lesion itself. Reported mortality rates remain between 30% and 36%, reinforcing the prognostic importance of systemic stability over local esophageal healing [2,5]. Follow-up endoscopy in surviving patients often shows complete mucosal recovery, though stricture formation may occur in a minority [9].

In our patient, serial endoscopy demonstrated full mucosal regeneration over months with conservative care and strict alcohol cessation. This favorable outcome reinforces that early recognition and appropriate supportive therapy can result in complete healing.

## Conclusions

AEN, or “black esophagus,” represents a rare but severe manifestation of upper GI injury often triggered by ischemic or chemical mucosal insult. In patients with a history of alcohol use, repeated vomiting, or transient hemodynamic instability, clinicians should maintain a high index of suspicion for this condition. In this case, the presence of esophageal varices and underlying cirrhosis significantly influenced management, including the need for banding. Although early endoscopic evaluation, aggressive supportive management, and strict avoidance of precipitating factors such as alcohol are important, it is worth noting that overall outcomes in AEN are largely determined by the severity of the underlying illness, and complete recovery, such as seen in this case, is uncommon, particularly in patients with liver disease. Awareness of this entity is essential, as timely diagnosis and treatment can significantly reduce morbidity and improve patient outcomes.
